# Supplementary material for: Translational buffering by ribosome stalling in upstream open reading frames
Source: PLoS Genet. 2022 Oct 31;18(10):e1010460. doi: 10.1371/journal.pgen.1010460 (PMC9648851; doi:10.1371/journal.pgen.1010460)
Supplement: Attachment — Submitted filename: response_letter.pdf [file pgen.1010460.s007.pdf]

Review Commons Full Revision

Manuscript number: RC-2022-01232

Corresponding authors: Adam P. Geballe, Arvind Rasi Subramaniam

Summary of changes

We thank all three reviewers for their constructive feedback on our manuscript. We have now perfomed extensive experiments, analyses, and rewriting of our manuscript to address all their concerns. We believe that these changes significantly improve the rigor of our conclusions and the clarity of our discussion. We highlight below key experiments, analyses, and re-writing in the revised manuscript, which is followed by a detailed point-by-point response.

1) We have now performed experiments using alternative uORF donor sequences to demonstrate the robustness of uORF repression to changes in uORF length.
2) By mutating out near-cognate start codons within uORF2, we have now demonstrated that near-cognate start codon initiation within uORF2 does not impact repression.
3) To quantify the dynamic range of our dual luciferase assay, we have now mutated out the NLuc start codon. We find that repressive uORF2 constructs have expression levels that are still > 20-fold above the no-start-codon control values.
4) We have now analyzed ribosome profiling coverage on uORFs (supplementary figure 5), and we show that several uORFs with known elongation stalls lack evidence of 40S and 80S subunit queueing 5′ to stalls, consistent with our collision-induced ribosome dissociation model.
5) We have now provided detailed discussion of footprint length choice in our modeling and the role of codon choice in our experiments.
6) We have now added a new main figure that provides a graphical representation of reactions considered in our kinetic modeling. This figure will make our modeling assumptions more transparent and accessible to readers with less computational expertise.

Reviewer 1

Summary

Bottorff et al test several models of uORF-mediated regulation of main ORF translation using the uORF2 of CMV UL4 gene, a system that has been previously experimentally characterized by the authors. They first train a computational model to recapitulate the observed experimental effects of mutations in uORF2, and then use the model to infer which uORF parameters may confer buffering against reduced ribosome loading that typically occurs upon biological perturbation. The authors then find that: i) the uORF2 confers buffering, ii) the uORF2 mechanism adjusts to computational predictions for the collision-mediated 40S dissociation model of uORF-mediated regulation.

Significance

This manuscript represents an interesting effort to distinguish mechanisms of uORF-mediated regulation based on mathematical modeling, and might be useful for the translation community.

My expertise: Regulation of translation.

We thank Reviewer 1 for a succinct summary of our main conclusions and highlighting the significance of our work to the translation community.

Major comments

1) Figure 4 (Figure 5 in revised version): Which is the dynamic range of the WT vs the no-stall construct? In the WT construct, main ORF translation is already quite repressed, and detecting further repression may be more difficult than in the no-stall construct. In other words, the differences that authors are detecting between the WT and no-stall constructs might be due to a potential lower dynamic range of the WT construct.

To measure the dynamic range of our reporter assay, we have now mutated the start codon of the NLuc reporter ORF. We reasoned that this construct provides a lower bound on measurable NLuc signal. The resulting no-NLuc-start-codon reporter expression was at least 20-fold lower than WT construct (Fig. S1A). Importantly, we also see that the raw NLuc signal of the WT construct is at least 20-fold over the background (Fig. S1B). Thus, the differential response of WT and no-stall constructs is not simply due to lower dynamic range of the WT construct.

2) The authors conclude that uORF2 follows the collision-mediated 40S dissociation model, based on fitness of their experimental results with predictions from their mathematical modeling regarding distance between uORF2 initiation codon and the stalling site. But can the authors actually directly prove that there are no 40S subunits accumulating behind the stalled 40S using Ribo-Seq or TCP-Seq?

We have now examined existing 80S Ribo-seq and 40S TCP-seq datasets to determine whether queued 40S or 80S ribosomes can be detected at known stall sites. Stern-Ginossar et al. (2012) performed 80S Ribo-seq during hCMV infection. In this dataset, while the stall at the *UL4* termination codon has a very high ribosome density, few elongating ribosomes are seen queued behind the stalled 80S, consistent with an absence of 80S ribosome queuing (Fig. RR1). By contrast, another well-studied elongation stall in the *Xbp1* mRNA shows ~30 nt periodic peaks in ribosome density indicative of ribosome queues (Fig. RR2). An important caveat is that queued ribosomes could be systematically underrepresented in standard Ribo-seq datasets due to incomplete nuclease digestion (Darnell et al., 2018; Subramaniam et al., 2014; Wolin and Walter, 1988).

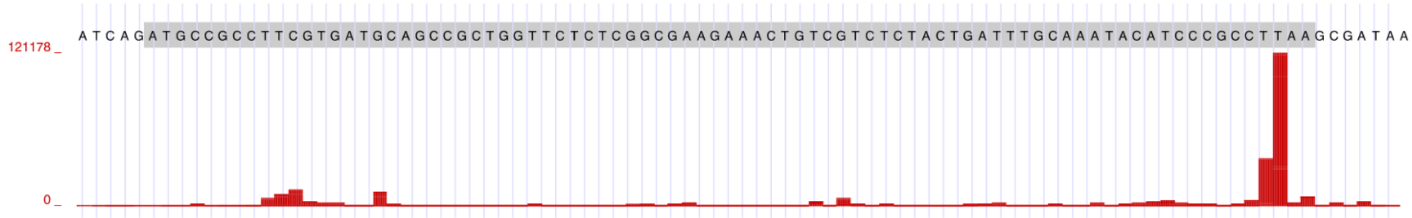

Figure RR1: Lack of elongating ribosome queueing at *UL4* uORF2 stall

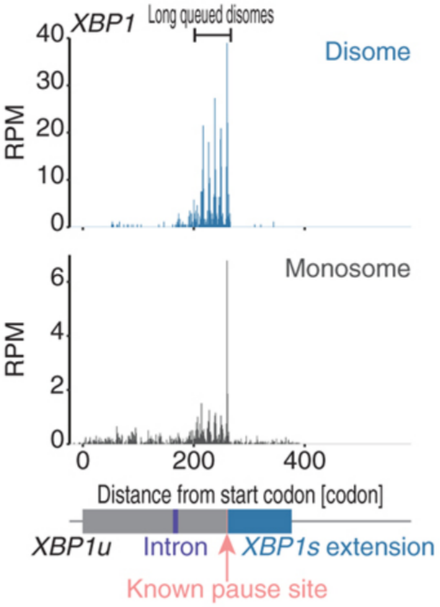

Figure RR2: Ribosome queueing at a known stall on Xbp1 mRNA (figure from (Han et al., 2020))

Since there is no 40S TCP-Seq dataset during hCMV infection, we examined other known stalls on human mRNAs (Fig. RR3 below; Fig. S5 in our manuscript). We examine small ribosomal subunit profiling data from human uORFs with conserved amino acid-dependent elongating ribosome stalls (Figure S5A). Ribosome density read

counts are low across all of these uORFs, showing no evidence of ribosome queuing. Subtle queues might not be observed given these low read counts from insufficient capture of small ribosomal subunits. Nevertheless, we do not observe any evidence of queueing upstream to elongating ribosome stalls in this data. We note these observations in our Discussion section as follows (lines 688-712): "Although our data from *UL4* uORF2 does not support the queuing-mediated enhanced repression model (Fig. 1C) (Ivanov et al., 2018), this model might describe translational dynamics on other mRNAs. Translation from near-cognate start codons is resistant to cycloheximide, perhaps due to queuing-mediated enhanced initiation, but sensitive to reductions in ribosome loading (Kearse et al., 2019). Loss of eIF5A, a factor that helps paused elongating ribosomes continue elongation, increases 5′ UTR translation in 10% of studied genes in human cells, augmented by downstream in-frame pause sites within 67 codons, perhaps also through queuing-mediated enhanced initiation (Manjunath et al., 2019). There is also evidence of queuing-enhanced uORF initiation in the 23 nt long *Neurospora crassa* arginine attenuator peptide (Gaba et al., 2020) as well as in transcripts with secondary structure near and 3′ to start codons (Kozak, 1989). Additional sequence elements in the mRNA might determine whether scanning ribosome collisions result in queuing or dissociation. Small subunit profiling data (Wagner et al., 2020) from human uORFs that have conserved amino acid-dependent elongating ribosome stalls do not show evidence of scanning ribosome queues (Fig. S5A), consistent with the collision-mediated 40S-dissociation model. Subtle queues might not be observed given these low read counts from insufficient capture of small ribosomal subunits."

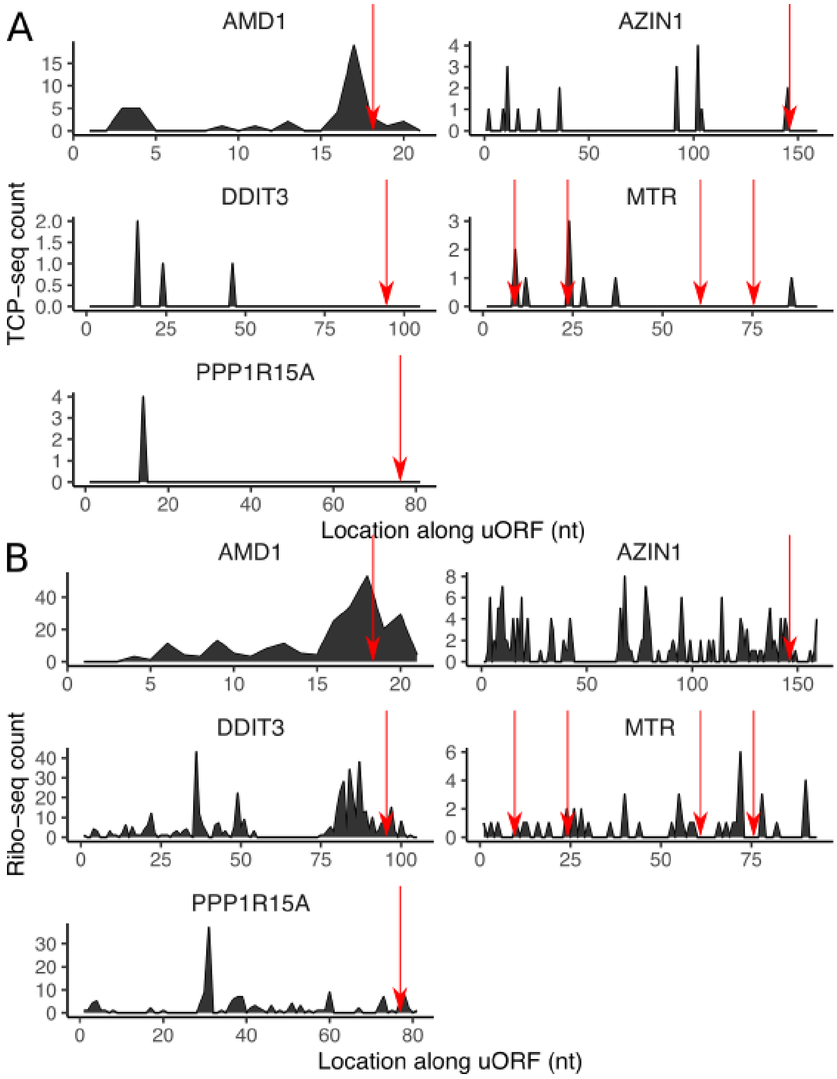

Figure RR3: Variable evidence of density 5′ to elongating ribosome stalls in uORFs (figure S5)

3) Experimental data in Figures 2, 4 and 5 include 3 technical replicates. Sound conclusions typically require biological replicates. Further, the number of replicates in Figure 6 has not been indicated.

As suggested by the reviewer, we have now included biological replicates for all luciferase assays [Figures 2, 5, 6, and 7 that were previously 2, 4, 5, and 6] that were technical replicates in the previous version. This replication does not alter any of our conclusions. We have now included the number of biological replicates for Figure 7 (former Figure 6).

Minor comments

1) Figure 4 (Figure 5 in revised version): It is strange that a PEST sequence had to be introduced in the construct of part B in order to observe reliable differences, but not in constructs of parts A and C. Can the authors explain?

We introduced the PEST sequence for part B because we wanted to measure the reporter response to treatment with a drug that reduces translation initiation. The PEST sequence increases the turnover rate of the reporter protein. Without the PEST sequence, the luminescence signal will be dominated by the reporter expression before the drug was added. However, in parts A and C, initiation rate was altered through genetic mutations and measuring their expression under basal conditions does not require a PEST sequence. Except in situations where a quick dynamic response needs to be measured such as in the drug treatment in part B, reporters without PEST sequences are simpler to interpret due to the absence of proteasome-mediated degradation and higher overall signal.

2) Figure 6 (Figure 7 in revised version): Unfortunately, the authors find no other human uORFs with terminal diproline motifs that are so essential for main ORF repression as uORF2. In this light, can the authors comment further on the usefulness of their findings for human genes? Have the authors searched for viral RNAs with similar features? Please, notice that the gene PPP1R37 has not been mentioned in the main text.

The *UL4* and human uORFs differ in their sequence determinants of translational repression. *UL4* uORF2 represses translation entirely through nascent peptide-mediated stalling. While the terminal diproline motif in *UL4* uORF2 is necessary for main ORF repression, it is not sufficient. A number of other residues in the *UL4* uORF2 peptide play a critical role in repression (Cao and Geballe, 1996; Matheisl et al., 2015). Thus, it is not surprising that human uORFs that we identified based solely on the presence of terminal diproline motifs confer only modest decrease in repression upon mutating the terminal proline. The human uORFs containing these terminal diprolines may partially repress translation via nascent peptide effects, but the majority of the repression likely arises from siphoning of scanning ribosomes from the main ORF (Fig. 1A in our manuscript) and inefficient termination following translation of consecutive prolines (Cao and Geballe, 1996; Cao and Geballe, 1998; Janzen et al., 2002; Matheisl et al., 2015). Our current understanding of features in nascent peptide that mediate translational repression (Wilson et al., 2016) is insufficient to bioinformatically identify elongation-stall containing uORFs in human or viral genomes, so we simply looked for terminal diprolines. Despite this limitiation, we note that the modeling approaches and experimental perturbations developed in our work can be applied to study ribosome kinetics on any repressive uORF, independent of the mRNA or peptide sequence underlying the repression.

As suggested by Reviewer 1, we have now included all the studied uORFs in the main text.

Reviewer 2

Summary

In this paper, the authors are exploring the uORF regulatory mechanism. They first discussed five general models how uORFs might work to repress and buffering main ORF translation, then they mainly focus on the UL4 uORF2 for the potential mechanism. They use both computer modeling and experimental validation with reporter assay in 293t cell line. Based on their model, and few experimental results when they change the translation initiation rate and/or length of dORF, they propose it may work through 40S dissociation model, since the buffering effect is not uORF length sensitive.

Significance

It is an interesting area, using modeling with experiment validation to understand uORF regulation mechanism, the kinetics and interplay between different translation steps, it will help us to understand uORF buffering in stress conditions. Also bring modeling method with reporter validation to the translation field, will provide clues to the molecular mechanism study, especially in complex situation.

We thank Reviewer 2 for a comprehensive summary of our work and noting the uniqueness and usefulness of our experiment-integrated modeling approach to the translation field.

Major comments

• Are the key conclusions convincing?

The modeling for different mechanisms is insightful, but some modeling parameters and experimental validation are not conclusive and validation of few of them can enforce the conclusions.

We have now performed key validation experiments suggested by Reviewer 2, notably: 1. mutating out of near-cognate start codons in the UL4 uORF2 coding sequence and 2. increasing UL4 uORF2 length using two unrelated protein coding sequences. Please see responses to specific comments below for further details.

• Should the authors qualify some of their claims as preliminary or speculative, or remove them altogether?

Yes, the part about queuing and length sensitive is not convincing to me, it should be modified and reduce the statement strength.

We agree about reducing the statement strength and have altered our statements as suggested by the reviewer. Specifically, we have now expanded the rationale for the choice of footprint lengths of 40S subunits. Please see responses to specific comments below for further details.

• Would additional experiments be essential to support the claims of the paper? Request additional experiments only where necessary for the paper as it is, and do not ask authors to open new lines of experimentation.

Yes, please see the specific concerns

• Are the suggested experiments realistic in terms of time and resources? It would help if you could add an estimated cost and time investment for substantial experiments.

They will need to re-think about the modeling, and validation in Figure 5, there are validation experiments that can be done in weeks and in a cost-efficient manner that can enforce the conclusions.

We have performed the experiments suggested by the reviewer. See responses below.

• Are the data and the methods presented in such a way that they can be reproduced?

Most of them are good

• Are the experiments adequately replicated and statistical analysis adequate?

Yes

Specific concerns

1) It is a bit confusing to me in Figure 2C, the reporter assays, that non-start codon reporter and non-stall reporter has same expression level. In theory, the non-stall reporter still has uORF there, so it should repress main ORF expression, and have lower expression level than the non-start reporter, where there is no uORF, no repression. In other uORFs they tested in Figure 6 (Figure 7 in revised version), the non-stall reporters are lower than non-start reporter. Since data they use to build the model is Figure 2B, and calculate the parameters for the whole paper, I just want to make sure it is making sense. I noticed there is another CTG in frame on the 4th codon, this may be alternative start codon in the non-start reporter to trigger some repression.

To address Reviewer 2's concern about alternative start codon usage in the non-start reporter, we have now mutated out all near-cognate start codons known to initiate translation with high frequency (Kearse and Wilusz, 2017). These near-cognate start codons consisted of Leu4 CTG, Leu11 CTG, Leu14 TTG, and Leu15 CTG and were mutated to CTA, CTA, TTA, and CTA, respectively. We find that removing the uORF2 near-cognate start codons does not significantly alter NLuc expression (Fig. S1A). This experiment merely rules out one possible source of these similar expression levels. We expect that uORF2 no-start and no-stall reporters' very similar NLuc expression levels can be rationalized for the several following reasons:

1. uORF2 initiation frequency is quite low. We estimate it to be 5% or less in our modeling based on previous measurements (Cao and Geballe, 1995). Thus, the maximum theoretically possible difference in NLuc expression between no-start and no-stall reporters is 5% or less.

2. Further, re-initiation after uORF2 translation is frequent. We estimate it to be around 50% within our manuscript, which will further decrease repression in the no-stall mutant. Thus, we expect the no-stall mutant to decrease the flux of scanning ribosomes at the main ORF by 2-3% compared to the no-start mutant.
3. Finally, a subtle but important point to note is that our reporter assays are measuring NLuc expression and not the flux of scanning ribosomes at the main ORF NLuc start codon. Since NLuc ORF has a strong start codon context (GCCACC) and the flux of scanning ribosomes is already high for the no-start and no-stall mutants, slight changes in the flux of scanning ribosomes are unlikely to impact NLuc expression. This is because start codon selection is not rate-limiting for protein expression under these conditions. This last point is clearly seen in high throughput reporter assays where the mutations which impact reporter expression in a non-optimal context have little or no effect in an optimal context (see Fig. 5B, 5C in Noderer et al., 2014).

Thus, in summary, even if the flux of scanning ribosomes is decreased by 3-5% by the no-stall uORF2 mutant compared to the no-start uORF2 mutant, we expect the effect on NLuc expression to be negligible and below the limit of our experimental resolution (which is ~10% based on the standard error across technical replicates).

Regarding the different behavior of the human uORFs in our manuscript and *UL4* uORF2, note the response to Reviewer 1 regarding the usefulness of our human uORF findings.

2) All the modeling and prediction the authors do are based on average, but we know translation is very heterogeneous. For each ribosome or each 40S, the kinetics varies a lot, the authors should discuss about this part.

We now discuss translation heterogeneity in the Discussion section in lines 781-794 as follows: "Translation heterogeneity among isogenic mRNAs has been observed in several single molecule translation studies (Boersma et al., 2019; Morisaki et al., 2016; Wang et al., 2020; Wu et al., 2016; Yan et al., 2016). This heterogeneity may arise from variability in intrasite RNA modifications (Yu et al., 2018), RNA binding protein occupancy, or RNA localization. We do not capture these sources of heterogeneity in our modeling since the observables in our simulations are averaged over long simulated time scales and used to predict only bulk experimental measurements. However, our models studied here can readily extended through compartmentalized and state-dependent reaction rates (Harris et al., 2016) to account for the different sources of heterogeneity observed in single molecule studies."

3) For modeling related with the queuing-mediated model in Figure 1C. they use 30nt as the ribosome length to count the potential queuing to start codon. But 30nt is the 80S protected fragment with specific conformation. The protected fragment for 80S will change based on different status of ribosome conformation or elongation step. More importantly, for queuing, it is 40S, so they may have a different size. Based on previous 40S ribosome profiling (Archer, Stuart K., et al. Nature 535.7613 (2016): 570-574. And other papers), the length can vary from 19nt to very long, so I don't think the 30nt length can be used to model queuing in 40S and length sensitivity in the uORF working mechanism.

We thank Reviewer 2 for highlighting this issue of footprint length heterogeneity that we had not previously addressed. In our modeling, we assume homogenous ribosome footprints. While, heterogeneous ribosome footprints have been observed for small ribosomal subunits (Bohlen et al., 2020; Wagner et al., 2020; Young et al., 2021) and elongating ribosomes (Lareau et al., 2014; Wu et al., 2019), we believe that our modeling of homogenous footprint length is appropriate for the following three reasons:

First, with respect to the small ribosomal subunit footprint heterogeneity, we note that TCP-seq studies include crosslinking of eukaryotic initiation factors (eIFs). The presence of these eIFs is thought to be the main source of heterogeneity in scanning ribosome footprints (Bohlen et al., 2020; Wagner et al., 2020). Although crosslinking is often performed, it is not necessary to obtain scanning ribosome footprints, and homogenous 30 nt footprints are observed in the absence of crosslinking (Bohlen et al., 2020). Notably, figure S2 of Bohlen et al. (2020), reproduced as Fig. RR4 below, shows that scanning SSU footprint lengths are tightly distributed around 30 nt when crosslinking is not used.

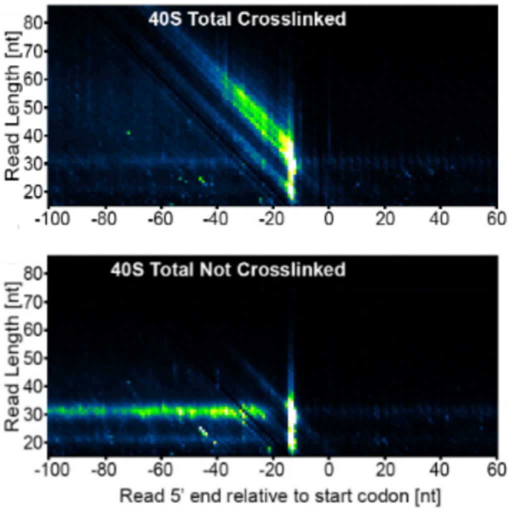

Figure RR4: Effect of crosslinking on scanning footprint heterogeneity (reproduced from Bohlen et al. (2020))

Second, in the context of the strong, minutes-long *UL4* uORF2 elongating ribosome stall (Cao and Geballe, 1998), collided ribosomes will wait for long periods of time relative to normal elongating or scanning ribosomes. Thus, we expect that associated eIFs dissociate from these dwelling ribosomes as they typically do during start codon selection or during translation of short uORFs (Bohlen et al., 2020).

Third, a significant fraction of mRNAs exhibit cap-tethered translation in which eIFs must dissociate from ribosomes before new cap-binding events, and therefore collisions, can occur (Bohlen et al., 2020).

Based on above three points, we believe that modeling the footprint of only the scanning ribosomes, and not the associated eIFs, using a single 30 nt length is biologically reasonable.

Footprint length heterogeneity of elongating ribosomes is much less drastic than that observed for scanning ribosomes and likely arises from different conformational states such as an empty or occupied A site (Lareau et al., 2014; Wu et al., 2019). While the different elongating ribosome footprints arise from differences in mRNA accessibility to nucleases, it is unclear whether the distance between two collided ribosomes changes across different ribosome conformations. For instance, the queues of elongating ribosomes observed at the Xbp1 mRNA stall occur at regular ~30 nt periodicity (Fig. RR2). Additionally, the stalled elongating ribosome is stuck in a pre-translocation state and has a defined, ~30 nt footprint (Wu et al., 2019), which only leaves room for 1 5′ queued ribosome within *UL4* uORF2 whose footprint is conformation sensitive.

Finally, a small degree of scanning footprint heterogeneity is also accounted for by our modeling of backward scanning which effectively introduces heterogeneity to collided scanning ribosome location on mRNAs (Figures 6A, S2D in our manuscript).

We have now summarized the above points in the Discussion section of the revised manuscript (lines 713-740).

4) For Figure 5B (Figure 6B in revised version), besides the modeling length part I have mentioned above, when the authors increase the length of uORF, the sequence is also changed, which may introduce other side effect. So, if the authors want to conclude about the queuing part, they should rethink about the length for both modeling and validation, plus control for the sequence they added to increase the length of uORF, for example use different sequence when manipulate the length.

As suggested by the Reviewer, we have now varied the length of uORF2 using a different, unrelated donor sequence encoding the FLAG peptide and observe similar results (Fig. S4 in our manuscript) to our original experiment with the YFP-encoding sequence (Fig. 6B in our manuscript). A slight trend towards derepression with longer uORFs is observed in both cases. This effect might arise due to decreased stall strength caused by higher nascent peptide protrusion out of the exit tunnel leading to cotranslational folding (Bhushan et al., 2010; Nilsson et al., 2015; Wilson et al., 2016) or nascent chain factors (Gamerdinger et al., 2019; Weber et al., 2020)

exerting a pulling force on the peptide. Importantly, we do not see the periodic change in repression predicted by the queueing model (Figure 6A, yellow-green lines).

Minor comments

• Specific experimental issues that are easily addressable.

5) It is unclear how the luciferase assays were analyzed considering the background noise. If the NLuc expression is low, close to the background, then how to extract or normalize the background will influence the expression level, thus fold change for different reporter/condition.

To account for the luciferase background, we subtracted background from measured data values. To show that expression is rarely close to background (from mock transfections), we included a supplementary figure showing raw NLuc and FLuc values (Fig. S1B). Also note the response to Reviewer 1 regarding a no-start-codon control having a 20-fold lower signal than the WT UL4 uORF2 construct.

• Are prior studies referenced appropriately?

yes

• Are the text and figures clear and accurate?

Mostly good

• Do you have suggestions that would help the authors improve the presentation of their data and conclusions?

Have a main figure about the modeling part.

As suggested by the Reviewer, we have now added visual representations of the reactions as a new main figure (Fig. 3). We also moved the modeling workflow figure from the supplementary set of figures to this main figure (Fig. 3). We thank the reviwer for this suggestion that greatly improves the presentation of our modeling methodology.

• Place the work in the context of the existing literature (provide references, where appropriate).

Recent years, there has been a lot of study about small open reading frames, while for uORFs are known to repress translation, the regulatory mechanism is not known yet, there are just different models not validated yet (Young & Wek, 2016). Also, under normal conditions and stress conditions, uORF can play both repressive and stimulative role in main ORF translation (Orr, Mona Wu, et al. NAR 48.3 (2020): 1029-1042.). This paper is the first study to put all the uORF working hypothesis with buffering effect together, they use modeling to explain how under each hypothesis, buffering may happen or not.

• State what audience might be interested in and influenced by the reported findings.

It will be interesting to people, who study molecular biology, biochemistry for translation regulation, especially uORFs. The modeling people may also find it interesting, how they could adapt modelinbeew keywords to help the authors contextualize your point of view. Indicate if there are any parts of the paper that you do not have sufficient expertise to evaluate. I have extensive experience working in the translation regulation field and I feel extremely comfortable to discus all the experimental part including individual reporters as well as genome wide. But I do not consider an expert in the modelling section of this work.

Reviewer 3

Summary

Small ORFs are prevalent in eukaryotic genomes with variety of functions. Recent technological advances enable their detection, yet our understanding on the mode of action remains quite rudimentary. The manuscript by Bottorff, Geballe and Subramaniam aims at elucidating the function of UL4 uORF in the CMV, and thus, it is on timely and topical research. The authors measure the uORF -controlled expression of the well-studies UL4 uORF and kinetically model the initiation behavior. Within a second uORF, a diproline pair controls initiation of the downstream main ORF sensing ribosomal collisions between a scanning small subunit and an 80S positioned at the canonical start of the main ORF. The stalling at both proline codons is envisioned as a kinetic window to sense any elongation-competent 80S at initiation and thus, control the ribosomal load and expression. Such diproline tandems are present in some uORFs in human transcriptome, hence representing more pervasive control mechanism.

Significance

I am unable to comment in depth on the modeling algorithms and simulations as this is outside of my expertise. The experiments are reasonably designed to test various models of uORF regulation and set the frame for the modelling. The idea that various stress factors would decrease canonical initiation and consequently would reflect the number of initiating ribosomes are adequately tested by varying the number of initiating ribosomes. The discovery of the two terminal prolines, that are also found in other human uORFs, is appealing mode of controlling stalling-driven downstream initiation. However, the lack of experimental support with the human uORFs may indicate additional contributions. This raises the question as to whether the proline codon identity plays a role? Since codons are read with different velocity which is mirrored by the tRNA concentration.

It would be good to address whether special proline codons have been evolutionarily selected in CMV and whether the kinetics of stalling strongly depends on the codon identity. Are both prolines in the tandem using the same codon? Along that line, are the same proline codons used in the human diproline-containing counterparts? Consequently, the P to A mutation may have altered the codon usage and could be the reason for the nonlinear effect in the human sequenced. In this case, it would make sence to use Ala-codons with similar codon usage as the natural prolines?

We thank the Reviewer for raising this point about the role of codon usage. The tandem proline residues do not use the same codon (CCG then CCT). The two C-terminal proline residues in uORF2 are necessary for the elongating ribosome stall (Bhushan et al., 2010; Degnin et al., 1993; Wilson et al., 2016), but it has been previously shown that the identity of the codon does not significantly impact repression (Degnin et al., 1993). The human uORFs generally have 1 of the 2 Pro codons in common with the uORF2 Pro codons. Given that most of the human uORF P to A mutations behave similarly (Figure 7) irrespective of the original proline codon, we believe that codon usage does not impact repression by these uORFs. Moreover, as explained in response to Reviewer 1 and 2's questions, we believe that the human uORFs containing terminal diprolines may partially repress translation via nascent peptide effects, but the majority of the repression likely arises from efficient siphoning of scanning ribosomes from the main ORF by the uORF (Fig. 1A in our manuscript).

References

Bhushan, S., Meyer, H., Starosta, A.L., Becker, T., Mielke, T., Berninghausen, O., Sattler, M., Wilson, D.N., and Beckmann, R. (2010). Structural Basis for Translational Stalling by Human Cytomegalovirus and Fungal Arginine Attenuator Peptide. Molecular Cell *40*, 138–146.

Boersma, S., Khuperkar, D., Verhagen, B.M.P., Sonneveld, S., Grimm, J.B., Lavis, L.D., and Tanenbaum, M.E. (2019). Multi-Color Single-Molecule Imaging Uncovers Extensive Heterogeneity in mRNA Decoding. Cell *178*, 458–472.e19.

Bohlen, J., Fenzl, K., Kramer, G., Bukau, B., and Teleman, A.A. (2020). Selective 40S Footprinting Reveals Cap-Tethered Ribosome Scanning in Human Cells. Molecular Cell *79*, 561–574.e5.

Cao, J., and Geballe, A.P. (1995). Translational inhibition by a human cytomegalovirus upstream open reading frame despite inefficient utilization of its AUG codon. J Virol *69*, 1030–1036.

Cao, J., and Geballe, A.P. (1996). Coding sequence-dependent ribosomal arrest at termination of translation. Molecular and Cellular Biology *16*, 603–608.

Cao, J., and Geballe, A.P. (1998). Ribosomal release without peptidyl tRNA hydrolysis at translation termination in a eukaryotic system. RNA *4*, 181–188.

Darnell, A.M., Subramaniam, A.R., and O'Shea, E.K. (2018). Translational Control through Differential Ribosome Pausing during Amino Acid Limitation in Mammalian Cells. Molecular Cell *71*, 229–243.e11.

Degnin, C., Schleiss, M., Cao, J., and Geballe, A. (1993). Translational inhibition mediated by a short upstream open reading frame in the human cytomegalovirus gpUL4 (gp48) transcript. Journal of Virology.

Gaba, A., Wang, H., Fortune, T., and Qu, X. (2020). Smart-ORF: a single-molecule method for accessing ribosome dynamics in both upstream and main open reading frames. Nucleic Acids Research.

Gamerdinger, M., Kobayashi, K., Wallisch, A., Kreft, S.G., Sailer, C., Schlömer, R., Sachs, N., Jomaa, A., Stengel, F., Ban, N., et al. (2019). Early Scanning of Nascent Polypeptides inside the Ribosomal Tunnel by NAC. Mol Cell *75*, 996–1006.e8.

Han, P., Shichino, Y., Schneider-Poetsch, T., Mito, M., Hashimoto, S., Udagawa, T., Kohno, K., Yoshida, M., Mishima, Y., Inada, T., et al. (2020). Genome-wide Survey of Ribosome Collision. Cell Reports *31*, 107610.

Harris, L.A., Hogg, J.S., Tapia, J.-J., Sekar, J.A.P., Gupta, S., Korsunsky, I., Arora, A., Barua, D., Sheehan, R.P., and Faeder, J.R. (2016). BioNetGen 2.2: advances in rule-based modeling. Bioinformatics *32*, 3366–3368.

Ivanov, I.P., Shin, B.-S., Loughran, G., Tzani, I., Young-Baird, S.K., Cao, C., Atkins, J.F., and Dever, T.E. (2018). Polyamine Control of Translation Elongation Regulates Start Site Selection on the Antizyme Inhibitor mRNA via Ribosome Queuing. Mol Cell *70*, 254–264.e6.

Janzen, D.M., Frolova, L., and Geballe, A.P. (2002). Inhibition of translation termination mediated by an interaction of eukaryotic release factor 1 with a nascent peptidyl-tRNA. Mol Cell Biol *22*, 8562–8570.

Kearse, M.G., and Wilusz, J.E. (2017). Non-AUG translation: a new start for protein synthesis in eukaryotes. Genes Dev *31*, 1717–1731.

Kearse, M.G., Goldman, D.H., Choi, J., Nwaezeapu, C., Liang, D., Green, K.M., Goldstrohm, A.C., Todd, P.K., Green, R., and Wilusz, J.E. (2019). Ribosome queuing enables non-AUG translation to be resistant to multiple protein synthesis inhibitors. Genes Dev *33*, 871–885.

Kozak, M. (1989). Circumstances and mechanisms of inhibition of translation by secondary structure in eucaryotic mRNAs. Mol Cell Biol *9*, 5134–5142.

Lareau, L.F., Hite, D.H., Hogan, G.J., and Brown, P.O. (2014). Distinct stages of the translation elongation cycle revealed by sequencing ribosome-protected mRNA fragments. eLife *3*, e01257.

Manjunath, H., Zhang, H., Rehfeld, F., Han, J., Chang, T.-C., and Mendell, J.T. (2019). Suppression of Ribosomal Pausing by eIF5A Is Necessary to Maintain the Fidelity of Start Codon Selection. Cell Reports *29*, 3134–3146.e6.

Matheisl, S., Berninghausen, O., Becker, T., and Beckmann, R. (2015). Structure of a human translation termination complex. Nucleic Acids Res *43*, 8615–8626.

Morisaki, T., Lyon, K., DeLuca, K.F., DeLuca, J.G., English, B.P., Zhang, Z., Lavis, L.D., Grimm, J.B., Viswanathan, S., Looger, L.L., et al. (2016). Real-time quantification of single RNA translation dynamics in living cells. Science *352*, 1425–1429.

Nilsson, O.B., Hedman, R., Marino, J., Wickles, S., Bischoff, L., Johansson, M., Müller-Lucks, A., Trovato, F., Puglisi, J.D., O'Brien, E.P., et al. (2015). Cotranslational Protein Folding inside the Ribosome Exit Tunnel. Cell Reports *12*, 1533–1540.

Noderer, W.L., Flockhart, R.J., Bhaduri, A., Diaz de Arce, A.J., Zhang, J., Khavari, P.A., and Wang, C.L. (2014). Quantitative analysis of mammalian translation initiation sites by FACS-seq. Mol Syst Biol *10*, 748.

Stern-Ginossar, N., Weisburd, B., Michalski, A., Le, V.T.K., Hein, M.Y., Huang, S.-X., Ma, M., Shen, B., Qian, S.-B., Hengel, H., et al. (2012). Decoding Human Cytomegalovirus. Science *338*, 1088–1093.

Subramaniam, Arvind R., Zid, Brian M., and O'Shea, Erin K. (2014). An Integrated Approach Reveals Regulatory Controls on Bacterial Translation Elongation. Cell *159*, 1200–1211.

Wagner, S., Herrmannová, A., Hronová, V., Gunišová, S., Sen, N.D., Hannan, R.D., Hinnebusch, A.G., Shirokikh, N.E., Preiss, T., and Valášek, L.S. (2020). Selective Translation Complex Profiling Reveals Staged Initiation and Co-translational Assembly of Initiation Factor Complexes. Mol Cell *79*, 546–560.e7.

Wang, H., Sun, L., Gaba, A., and Qu, X. (2020). An in vitro single-molecule assay for eukaryotic cap-dependent translation initiation kinetics. Nucleic Acids Res *48*, e6.

Weber, R., Chung, M.-Y., Keskeny, C., Zinnall, U., Landthaler, M., Valkov, E., Izaurralde, E., and Igreja, C. (2020). 4EHP and GIGYF1/2 Mediate Translation-Coupled Messenger RNA Decay. Cell Reports *33*, 108262.

Wilson, D.N., Arenz, S., and Beckmann, R. (2016). Translation regulation via nascent polypeptide-mediated ribosome stalling. Current Opinion in Structural Biology *37*, 123–133.

Wolin, S.L., and Walter, P. (1988). Ribosome pausing and stacking during translation of a eukaryotic mRNA. EMBO J *7*, 3559–3569.

Wu, B., Eliscovich, C., Yoon, Y.J., and Singer, R.H. (2016). Translation dynamics of single mRNAs in live cells and neurons. Science *352*, 1430–1435.

Wu, C.C.-C., Zinshteyn, B., Wehner, K.A., and Green, R. (2019). High-Resolution Ribosome Profiling Defines Discrete Ribosome Elongation States and Translational Regulation during Cellular Stress. Molecular Cell *73*, 959–970.e5.

Yan, X., Hoek, Tim A., Vale, Ronald D., and Tanenbaum, Marvin E. (2016). Dynamics of Translation of Single mRNA Molecules In Vivo. Cell *165*, 976–989.

Young, D.J., Meydan, S., and Guydosh, N.R. (2021). 40S ribosome profiling reveals distinct roles for Tma20/Tma22 (MCT-1/DENR) and Tma64 (eIF2D) in 40S subunit recycling. Nat Commun *12*, 2976.

Yu, J., Chen, M., Huang, H., Zhu, J., Song, H., Zhu, J., Park, J., and Ji, S.-J. (2018). Dynamic m6A modification regulates local translation of mRNA in axons. Nucleic Acids Research *46*, 1412–1423.
